# 2-Acetamido-3-(4-hydr­oxy-3-methoxy­phen­yl)acrylic acid

**DOI:** 10.1107/S1600536808001347

**Published:** 2008-02-06

**Authors:** Xiao-Bing Lan, Ye-Fei Nan, Lin Chen, Shi-Xiang Wang, Xiao-Hui Zheng

**Affiliations:** a School of Chemistry and Biotechnology, Yunnan Nationalities University, Kunming 650031, People’s Republic of China; b Engineering and Research Center of Chinese Herb Modernization, Northwest University, Xi’an 710069, People’s Republic of China

## Abstract

In the title compound, C_12_H_13_NO_5_, the azlactone of vanillin, the acrylic acid side chain has a *trans* extended conformation. There are inter­molecular N—H⋯O and O—H⋯O hydrogen bonds in the crystal structure.

## Related literature

For a related structure, see: Haasbroek *et al.* (1998 ▶). For information on the synthesis, see: Wong *et al.* (1992 ▶).
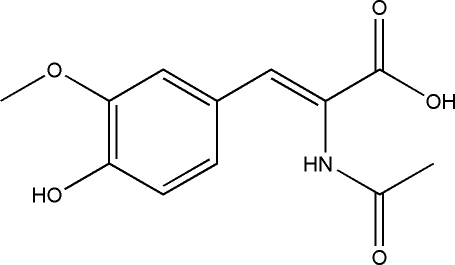


## Experimental

### 

#### Crystal data

C_12_H_13_NO_5_
*M* *_r_* = 251.23
Orthorhombic, 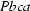

*a* = 12.7573 (14) Å
*b* = 12.7518 (14) Å
*c* = 14.7290 (17) Å
*V* = 2396.1 (5) Å^3^
*Z* = 8
Mo *K*α radiation
μ = 0.11 mm^−1^
*T* = 296 (2) K
0.37 × 0.30 × 0.26 mm

#### Data collection

Bruker SMART CCD area-detector diffractometer
Absorption correction: none
11011 measured reflections
2122 independent reflections
1788 reflections with *I* > 2σ(*I*)
*R*_int_ = 0.021

#### Refinement

*R*[*F* ^2^ > 2σ(*F* ^2^)] = 0.037
*wR*(*F* ^2^) = 0.122
*S* = 1.01
2122 reflections
167 parameters
H-atom parameters constrained
Δρ_max_ = 0.20 e Å^−3^
Δρ_min_ = −0.31 e Å^−3^

### 

Data collection: *SMART* (Bruker, 1997 ▶); cell refinement: *SAINT* (Bruker, 1997 ▶); data reduction: *SAINT*; program(s) used to solve structure: *SHELXS97* (Sheldrick, 2008 ▶); program(s) used to refine structure: *SHELXL97* (Sheldrick, 2008 ▶); molecular graphics: *SHELXTL* (Sheldrick, 2008 ▶); software used to prepare material for publication: *SHELXTL*.

## Supplementary Material

Crystal structure: contains datablocks I, global. DOI: 10.1107/S1600536808001347/cf2176sup1.cif

Structure factors: contains datablocks I. DOI: 10.1107/S1600536808001347/cf2176Isup2.hkl

Additional supplementary materials:  crystallographic information; 3D view; checkCIF report

## Figures and Tables

**Table 1:** Hydrogen-bond geometry (Å, °)

*D*—H⋯*A*	*D*—H	H⋯*A*	*D*⋯*A*	*D*—H⋯*A*
O4—H4⋯O3^i^	0.82	1.79	2.6044 (15)	171
O1—H1*B*⋯O5^ii^	0.82	1.84	2.6582 (13)	177
N1—H1⋯O1^iii^	0.86	2.13	2.9501 (14)	159

Symmetry codes: (i) 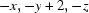; (ii) 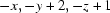; (iii) 
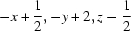
.

**Fig. 1.**
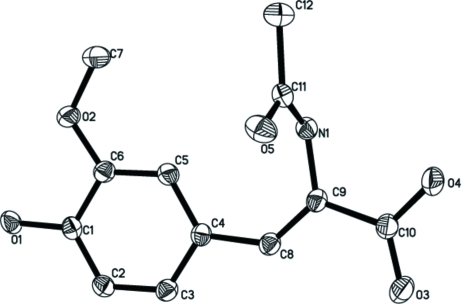
The molecular structure of (I), with atom labels and 30% probability displacement ellipsoids for non-H atoms. H atoms have been omitted.

**Fig. 2.**
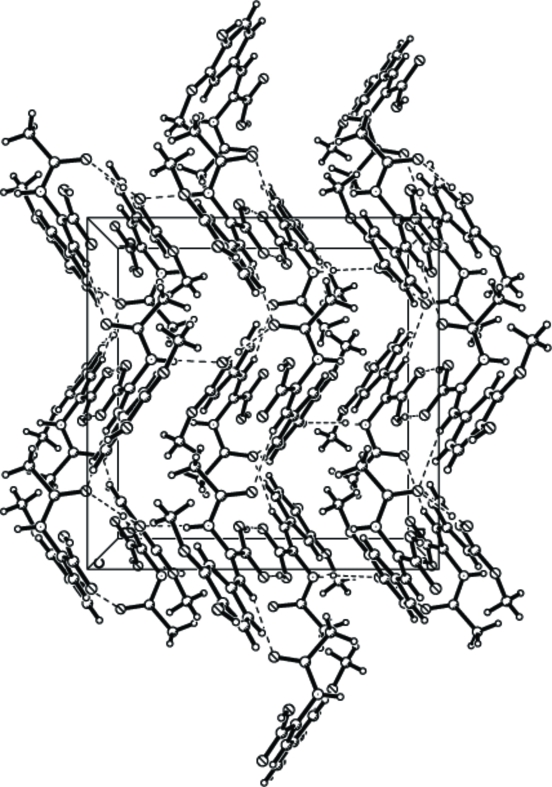
The packing of (I), viewed down the c axis. Molecules are connected by O—H···O and N—H···O hydrogen bonds shown as dashed lines.

## supplementary crystallographic information

### Comment

The molecular structure of the title compound is illustrated in Fig. 1. There are three intermolecular hydrogen bonds. One is formed between the NH group and the phenolic hydroxyl O atom of another molecule, the others between the carbonyl O atom and OH group.

A similar structure has been reported for a related compound (Haasbroek *et al.*, 1998).

### Experimental

The azlactone (Wong *et al.*, 1992) of vanillin (2.0 g, 7.3 mmol) was heated at 353 K with stirring in a sodium hydroxide solution (20 ml, 20%). A dark wine-red solution formed. After 3 h, the mixture was cooled and acidified with hydrochloric acid (6 *M*) to a pH of 1.35. The mixture was stirred well and allowed to cool to 273 K. The crystals that formed were filtered off and dried under vacuum at 318 K (1.45 g, 5.7 mmol). Recrystallization from ethanol/water gave pale-yellow crystals of (I) with a melting point of 481 K. Yellow single crystals suitable for X-ray analysis were obtained by slow evaporation of a solution in methanol–water at room temperature for two weeks. Spectroscopic analysis: IR (KBr, cm^-1^): 3256, 2952, 1678, 1656; ^1^H NMR (DMSO, δ, p.p.m.): 12.389 (s, 1 H), 9.492 (s, 1 H), 9.316 (s, 1 H), 7.286–7.282 (d, 1 H), 7.196 (s, 1 H), 7.088–7.068 (m, 1 H), 6.797–6.781 (d, 1 H), 3.771 (s, 3 H), 1.985 (s, 3 H).

### Refinement

H atoms bonded to N and O atoms were located in a difference map and refined as riding, with O—H = 0.82 and N—H = 0.86 Å, and with *U*_iso_(H) = 1.2*U*_eq_(O,*N*). Other H atoms were positioned geometrically and refined using a riding model, with C—H = 0.93–0.96 Å and *U*_iso_(H) = 1.2*U*_eq_(C) [1.5*U*_eq_(C) for methyl groups].

### Crystal data

**Table:**

C_12_H_13_NO_5_	*D*_x_ = 1.393 Mg m^−^^3^
*M**_r_* = 251.23	Melting point: 481 K
Orthorhombic, *P**b**c**a*	Mo *K*α radiation λ = 0.71073 Å
*a* = 12.7573 (14) Å	Cell parameters from 4984 reflections
*b* = 12.7518 (14) Å	θ = 2.7–28.1º
*c* = 14.7290 (17) Å	µ = 0.11 mm^−^^1^
*V* = 2396.1 (5) Å^3^	*T* = 296 (2) K
*Z* = 8	Block, yellow
*F*_000_ = 1056	0.37 × 0.30 × 0.26 mm

### Data collection

**Table:**

Bruker SMART CCD area-detector diffractometer	1788 reflections with *I* > 2σ(*I*)
Radiation source: fine-focus sealed tube	*R*_int_ = 0.021
Monochromator: graphite	θ_max_ = 25.1º
*T* = 296(2) K	θ_min_ = 2.7º
φ and ω scans	*h* = −15→13
Absorption correction: none	*k* = −15→15
11011 measured reflections	*l* = −14→17
2122 independent reflections	

### Refinement

**Table:**

Refinement on *F*^2^	Secondary atom site location: difference Fourier map
Least-squares matrix: full	Hydrogen site location: inferred from neighbouring sites
*R*[*F*^2^ > 2σ(*F*^2^)] = 0.037	H-atom parameters constrained
*wR*(*F*^2^) = 0.122	*w* = 1/[σ^2^(*F*_o_^2^) + (0.1*P*)^2^ + 0.0344*P*] where *P* = (*F*_o_^2^ + 2*F*_c_^2^)/3
*S* = 1.01	(Δ/σ)_max_ < 0.001
2122 reflections	Δρ_max_ = 0.20 e Å^−^^3^
167 parameters	Δρ_min_ = −0.31 e Å^−^^3^
Primary atom site location: structure-invariant direct methods	Extinction correction: none

### Special details

**Table:**

Geometry. All e.s.d.'s (except the e.s.d. in the dihedral angle between two l.s. planes) are estimated using the full covariance matrix. The cell e.s.d.'s are taken into account individually in the estimation of e.s.d.'s in distances, angles and torsion angles; correlations between e.s.d.'s in cell parameters are only used when they are defined by crystal symmetry. An approximate (isotropic) treatment of cell e.s.d.'s is used for estimating e.s.d.'s involving l.s. planes.
Refinement. Refinement of *F*^2^ against ALL reflections. The weighted *R*-factor *wR* and goodness of fit *S* are based on *F*^2^, conventional *R*-factors *R* are based on *F*, with *F* set to zero for negative *F*^2^. The threshold expression of *F*^2^ > σ(*F*^2^) is used only for calculating *R*-factors(gt) *etc*. and is not relevant to the choice of reflections for refinement. *R*-factors based on *F*^2^ are statistically about twice as large as those based on *F*, and *R*-factors based on ALL data will be even larger.

### Fractional atomic coordinates and isotropic or equivalent isotropic displacement parameters (Å^2^)

**Table:**

	*x*	*y*	*z*	*U*_iso_*/*U*_eq_	
N1	0.16380 (8)	0.89736 (8)	0.23064 (7)	0.0337 (3)	
H1	0.2301	0.9037	0.2218	0.040*	
O1	0.12427 (7)	1.09483 (8)	0.65078 (6)	0.0426 (3)	
H1B	0.0745	1.1269	0.6725	0.064*	
O2	0.22102 (8)	0.94162 (8)	0.56758 (7)	0.0473 (3)	
O3	−0.00149 (10)	1.06674 (9)	0.09716 (8)	0.0604 (4)	
O4	0.07449 (9)	0.91314 (8)	0.06704 (7)	0.0521 (3)	
H4	0.0520	0.9262	0.0161	0.078*	
O5	0.03350 (7)	0.79383 (7)	0.28136 (8)	0.0465 (3)	
C1	0.11343 (9)	1.08974 (10)	0.55890 (9)	0.0330 (3)	
C2	0.05408 (9)	1.16043 (10)	0.50975 (9)	0.0363 (3)	
H2	0.0209	1.2156	0.5394	0.044*	
C3	0.04374 (10)	1.14964 (10)	0.41675 (9)	0.0361 (3)	
H3	0.0033	1.1977	0.3846	0.043*	
C4	0.09273 (9)	1.06807 (9)	0.37029 (9)	0.0320 (3)	
C5	0.15561 (10)	0.99846 (10)	0.42060 (9)	0.0353 (3)	
H5	0.1912	0.9449	0.3908	0.042*	
C6	0.16531 (9)	1.00837 (10)	0.51307 (9)	0.0342 (3)	
C7	0.26213 (15)	0.85003 (13)	0.52600 (12)	0.0631 (5)	
H7A	0.2063	0.8115	0.4979	0.095*	
H7B	0.2952	0.8070	0.5712	0.095*	
H7C	0.3127	0.8696	0.4808	0.095*	
C8	0.07026 (10)	1.05762 (10)	0.27390 (9)	0.0339 (3)	
H8	0.0296	1.1117	0.2503	0.041*	
C9	0.09724 (9)	0.98457 (10)	0.21286 (9)	0.0326 (3)	
C10	0.05389 (11)	0.98977 (10)	0.12025 (9)	0.0383 (3)	
C11	0.12612 (9)	0.80575 (10)	0.26066 (9)	0.0344 (3)	
C12	0.20317 (13)	0.71764 (12)	0.27005 (12)	0.0547 (4)	
H12A	0.1975	0.6877	0.3296	0.082*	
H12B	0.2729	0.7439	0.2612	0.082*	
H12C	0.1884	0.6649	0.2253	0.082*	

### Atomic displacement parameters (Å^2^)

**Table:**

	*U*^11^	*U*^22^	*U*^33^	*U*^12^	*U*^13^	*U*^23^
N1	0.0316 (6)	0.0389 (6)	0.0306 (6)	0.0028 (4)	0.0014 (4)	−0.0005 (4)
O1	0.0364 (5)	0.0617 (7)	0.0295 (6)	0.0091 (4)	−0.0031 (4)	−0.0095 (4)
O2	0.0569 (6)	0.0501 (6)	0.0349 (6)	0.0194 (5)	−0.0089 (4)	−0.0023 (4)
O3	0.0912 (8)	0.0506 (7)	0.0393 (7)	0.0255 (6)	−0.0191 (6)	−0.0037 (5)
O4	0.0774 (8)	0.0472 (6)	0.0317 (6)	0.0116 (5)	−0.0112 (5)	−0.0069 (4)
O5	0.0448 (6)	0.0363 (6)	0.0583 (7)	−0.0007 (4)	0.0118 (5)	−0.0027 (4)
C1	0.0288 (6)	0.0413 (7)	0.0289 (7)	−0.0045 (5)	−0.0015 (5)	−0.0042 (5)
C2	0.0366 (6)	0.0353 (7)	0.0370 (8)	0.0011 (5)	0.0012 (5)	−0.0052 (6)
C3	0.0394 (7)	0.0332 (7)	0.0357 (8)	0.0009 (5)	−0.0019 (5)	0.0020 (5)
C4	0.0343 (6)	0.0315 (6)	0.0301 (7)	−0.0032 (5)	−0.0001 (5)	0.0011 (5)
C5	0.0373 (7)	0.0352 (7)	0.0333 (8)	0.0019 (5)	−0.0006 (5)	−0.0038 (5)
C6	0.0318 (6)	0.0368 (7)	0.0339 (8)	0.0006 (5)	−0.0040 (5)	−0.0001 (5)
C7	0.0809 (11)	0.0573 (10)	0.0512 (10)	0.0334 (9)	−0.0081 (9)	−0.0042 (8)
C8	0.0386 (6)	0.0314 (7)	0.0319 (8)	0.0002 (5)	−0.0011 (5)	0.0030 (5)
C9	0.0366 (7)	0.0326 (7)	0.0286 (7)	−0.0009 (5)	−0.0006 (5)	0.0036 (5)
C10	0.0490 (8)	0.0349 (7)	0.0310 (8)	0.0019 (5)	−0.0018 (6)	0.0001 (5)
C11	0.0404 (7)	0.0354 (7)	0.0275 (7)	0.0044 (5)	0.0010 (5)	−0.0042 (5)
C12	0.0614 (9)	0.0458 (9)	0.0570 (11)	0.0191 (7)	0.0032 (7)	0.0022 (7)

### Geometric parameters (Å, °)

**Table:**

N1—C11	1.3383 (17)	C3—H3	0.930
N1—C9	1.4236 (16)	C4—C5	1.4074 (18)
N1—H1	0.860	C4—C8	1.4544 (19)
O1—C1	1.3619 (17)	C5—C6	1.3734 (19)
O1—H1B	0.820	C5—H5	0.930
O2—C6	1.3690 (15)	C7—H7A	0.960
O2—C7	1.4192 (18)	C7—H7B	0.960
O3—C10	1.2563 (17)	C7—H7C	0.960
O4—C10	1.2798 (17)	C8—C9	1.3396 (19)
O4—H4	0.820	C8—H8	0.930
O5—C11	1.2298 (15)	C9—C10	1.4733 (19)
C1—C2	1.3819 (18)	C11—C12	1.4992 (19)
C1—C6	1.4037 (18)	C12—H12A	0.960
C2—C3	1.3831 (19)	C12—H12B	0.960
C2—H2	0.930	C12—H12C	0.960
C3—C4	1.3931 (18)		
			
C11—N1—C9	121.90 (10)	O2—C7—H7A	109.5
C11—N1—H1	119.0	O2—C7—H7B	109.5
C9—N1—H1	119.0	H7A—C7—H7B	109.5
C1—O1—H1B	109.5	O2—C7—H7C	109.5
C6—O2—C7	116.78 (11)	H7A—C7—H7C	109.5
C10—O4—H4	109.5	H7B—C7—H7C	109.5
O1—C1—C2	123.03 (11)	C9—C8—C4	131.93 (12)
O1—C1—C6	117.72 (11)	C9—C8—H8	114.0
C2—C1—C6	119.25 (12)	C4—C8—H8	114.0
C1—C2—C3	120.41 (12)	C8—C9—N1	124.96 (12)
C1—C2—H2	119.8	C8—C9—C10	119.58 (12)
C3—C2—H2	119.8	N1—C9—C10	115.43 (11)
C2—C3—C4	121.20 (12)	O3—C10—O4	123.11 (13)
C2—C3—H3	119.4	O3—C10—C9	119.79 (12)
C4—C3—H3	119.4	O4—C10—C9	117.10 (12)
C3—C4—C5	117.90 (12)	O5—C11—N1	122.34 (11)
C3—C4—C8	117.35 (11)	O5—C11—C12	120.96 (12)
C5—C4—C8	124.65 (11)	N1—C11—C12	116.69 (12)
C6—C5—C4	121.03 (12)	C11—C12—H12A	109.5
C6—C5—H5	119.5	C11—C12—H12B	109.5
C4—C5—H5	119.5	H12A—C12—H12B	109.5
O2—C6—C5	124.83 (12)	C11—C12—H12C	109.5
O2—C6—C1	114.99 (12)	H12A—C12—H12C	109.5
C5—C6—C1	120.16 (12)	H12B—C12—H12C	109.5
			
O1—C1—C2—C3	−178.29 (11)	C2—C1—C6—C5	−1.02 (18)
C6—C1—C2—C3	1.61 (18)	C3—C4—C8—C9	−173.93 (14)
C1—C2—C3—C4	−0.21 (19)	C5—C4—C8—C9	2.4 (2)
C2—C3—C4—C5	−1.73 (18)	C4—C8—C9—N1	−3.7 (2)
C2—C3—C4—C8	174.86 (11)	C4—C8—C9—C10	174.16 (13)
C3—C4—C5—C6	2.31 (18)	C11—N1—C9—C8	89.19 (16)
C8—C4—C5—C6	−174.01 (12)	C11—N1—C9—C10	−88.76 (15)
C7—O2—C6—C5	−6.86 (19)	C8—C9—C10—O3	5.4 (2)
C7—O2—C6—C1	171.24 (13)	N1—C9—C10—O3	−176.52 (13)
C4—C5—C6—O2	177.05 (11)	C8—C9—C10—O4	−173.86 (13)
C4—C5—C6—C1	−0.96 (19)	N1—C9—C10—O4	4.20 (18)
O1—C1—C6—O2	0.68 (16)	C9—N1—C11—O5	−5.8 (2)
C2—C1—C6—O2	−179.22 (11)	C9—N1—C11—C12	175.51 (12)
O1—C1—C6—C5	178.88 (11)		

### Hydrogen-bond geometry (Å, °)

**Table:**

*D*—H···*A*	*D*—H	H···*A*	*D*···*A*	*D*—H···*A*
O4—H4···O3^i^	0.82	1.79	2.6044 (15)	171
O1—H1B···O5^ii^	0.82	1.84	2.6582 (13)	177
N1—H1···O1^iii^	0.86	2.13	2.9501 (14)	159

Symmetry codes: (i) −*x*, −*y*+2, −*z*; (ii) −*x*, −*y*+2, −*z*+1; (iii) −*x*+1/2, −*y*+2, *z*−1/2.
